# Account of New Anæsthetic Agent, as a Substitute for Sulphuric Ether in Surgery and Midwifery

**Published:** 1848-02

**Authors:** 


*Account of a New Anæsthetic Agent, as a Substitute for Sulphuric Ether in Surgery and Midwifery.* Communicated to the Medico-chirurgical Society of Edinburgh, at their meeting on 10th Nov. 1847, by J. Y. Simpson, M.D., F.R.S.E., Prof. of Midwifery in the University of Edinburgh; Physician-Accoucheur to the Queen in Scotland, etc., etc.

From the time at which I first saw ether-inhalation successfully practiced in January last, I have had the conviction impressed upon my mind, that we would ultimately find that other therapeutic agents were capable of being introduced with equal rapidity and success into the system, through the same extensive and powerful channel of pulmonary absorption. In some observations, which I wrote and published in February last, relative to the inhalation of sulphuric ether in Midwifery, I stated that, in several obstetric cases, I had used ergot of rye in this way, along with ether.—(*See Monthly Journal of Medical Science*, p. 724.)

With various professional friends, more conversant with chemistry than I am, I have, since that time, taken oportunities of talking over the idea which I entertained of the probable existence or discovery of new therapeutic agents, capable of being introduced into the system by respiration, and the possibility of producing for inhalation vaporizable or volatile preparations of some of our more active and old established medicines : and I have had, during the summer and autumn, ethereal tinctures, &c., of several potent drugs, manufactured for me, for experiment, by Messrs. Duncan, Flockhart, & Co., the excellent chemists and druggists of this city.

Latterly, in order to avoid, if possible, some of the inconveniences and objections pertaining to sulphuric ether, (particularly its disagreeable and very persistent smell, its occasional tendency to irritation of the bronchi during its first inspirations, and the large quantity of it occasionally required to be used, more especially in protracted cases of labor,)—I have tried upon myself and others the inhalation of other volatile fluids, with the hope that some one of them might be found to possess the advantages of ether, without its disadvantages. For this purpose, I selected for experiment and have inhaled several chemical liquids of a more fragant or agreeable odor, such as the chloride of hydro-carbon (or Dutch liquid,) acetone, nitrate of oxide of ethyle (nitric ether,) benxin, the vapor of iodoform, &c.* I have found, however, one infinitely more efficacious than any of the others, viz: cloroform, or the perchloride of formyle, and I am enabled to speak most confidently of its superior anæsthetic properties, having now tried it upon upwards of thirty individuals. The liquid I have used has been manufactured for me by Mr. Hunter, in the laboratory of Messrs. Duncan, Flockhart & Co.

Chloroform was first discovered and described at nearly the same time by Soubeiran (1831,) and Liebig, (1832 ;) its composition was first accurately ascertained by the distinguished French chemist, Dumas, in 1835. See the *Annales de Chimie et de Physique*, vols. xlviii, xlix, and lviii. It has been used by some practitioners internally ; Guillot prescribed it as an antispasmodic in asthma, exhibiting it in small doses, and diluted 100 times. (See Bouchardat's *Annuaire de Therapeutique* for 1844, p. 35.) But no person, so far as I am aware, has used it by inhalation, or discovered its remarkable anæsthetic properties till the date of my own experiments.

It is a dense, limpid, colorless liquid, readily evaporating, and possessing an agreeable, fragrant, fruit-like odor, and a saccharine pleasant taste.

As an inhaled anæsthetic agent, it possesses over sulphuric ether the following advantages :

1. A greatly less quantity of chloroform than of ether is requisite to produce the anæsthetic effect ; usually from a hundred to a

*In talking over, with different chemists, what fluids might be sufficiently volatile to to be respirable, and hence deserving of being experimented upon, Mr. Waldie first named to me the perchloride of formyle as worthy, among others, of a trial; Dr. Gregory suggested a trial of the chloride of hydrocarbon, &c. I have been deeply indebted to Dr. Gregory and Dr. Anderson, for their kindness in furnishing me with the requisite chemical agents for these experiments; and also to my assistants, Dr. Keith and Dr. Duncan, for the great and hearty zeal with which they have constantly aided me in conducting the inquiry.

hundred and twenty drops of chloroform only being sufficient ; and with some patients much less. I have seen a strong person rendered completely insensible by six or seven inspirations of thirty drops of the liquid.

2. Its action is much more rapid and complete, and generally more persistent. I have almost always seen from ten to twenty full inspirations suffice. Hence the time of the surgeon is saved ; and that preliminary stage of excitement, which pertains to all narcotizing agents, being curtailed, or indeed practically abolished, the patient has not the same degree of tendency to exhilaration talking.*

3. Most of those who know from previous experience the sensations produced by ether inhalation, and who have subsequently breathed the chloroform, have strongly declared the inhalation and influence of chloroform to be far more agreeable and pleasant than those of ether.

4. I believe, that considering the small quantity requisite, as compared with ether, the use of chloroform will be less expensive than than of ether, more especially, as there is every prospect that the means of forming it may be simplified and cheapened.

5. Its perfume is not unpleasant, but the reverse ; and the odor of it does not remain, for any length of time, obstinately attached to the clothes of the attendant, or exhaling in a disagreeable form from the lungs of the patient, as so generally happens with sulphuric ether.

---

* In practice I have found that any such tendency, even with ether, is avoided by, 1st, giving the patient from the first a large and overwhelming dose of the vapor, and 2ndly, by keeping him perfectly quiet and still, and preventing all noise and talking around him. I have elsewhere insisted on the importance of these points. [See the numbers of the Monthly *Journal of Medical Science* for March, 1847, p. 726, and for September, p. 154.) In the paper last referred to, I took occasion, when discussing the conditions requisite for insuring successful etherization, to observe, " *First.*—The patient ought to be left, as far as possible, in a state of absolute quietude and freedom from mental excitement, both during the induction of etherization, and during his recovery from it. All talking and all questioning should be strictly prohibited. In this way any tendency to excitement is eschewed, and the proper effect of the ether inhalation more speedily and certainly induced. And, *Secondly*, with the same view, the primary stage of exhilaration should be entirely avoided, or at least reduced to the shortest possible limit, by impregnating the respired air as fully with the ether vapor as the patient can bear, and by allowing it to pass into the lungs both by the mouth and nostrils, so as rapidly and at once to superinduce its complete and anæsthetic effect; * * * * * a very common but certainly a very unpardonable error being to exhibit an imperfect and exciting, instead of a perfect and narcotizing dose of the vapor. Many of the alleged failures and misadventures are doubtless entirely attributable to the neglect of this simple rule ; not the principle of etherization, but the mode of putting it in practice being altogether to blame." But, *Thirdly*, whatever means or mode of etherization is adopted, the most important of the conditions required for procuring a satisfactory and successful result from its employment in surgery, consists in obstinately determining to avoid the commencement of the operation itself, and never venturing to apply the knife *until* the patient is under the influence of the ether-vapor, and "*thoroughly and indubitably soporized by it.*" In fulfilling all these indications, the employment of chloroform evidently offers great and decided advantages, in facility and efficiency, over the employment of ether.

6. Being required in much less quantity, it is much more portable and transmissible than sulphuric ether.

7. No special kind of inhaler or instrument is necessary for its exhibition. A little of the liquid diffused upon the interior of a hollow-shaped sponge, or a pocket-handkerchief, or a piece of linen or paper, and held over the mouth and nostrils, so as to be fully inhaled, generally suffices in about a minute or two to produce the desired effect.*

I have not yet had an opportunity of using chloroform in any capital surgical operation, but have exhibited it with perfect success in tooth-drawing,† opening abscesses, for annulling the pain of dysmenorrhœa of neuralgia, and in two or three cases where I was using deep, and otherwise very painful galvanopuncture for the treatment of ovarian dropsy, &c. I have employed it also in obstretric practice with entire success. The lady to whom it was first exhibited during parturition, had been previously delivered in the country by perforation of the head of the infant, after a labor of three days' duration. In this, her second confinement, pains superventeda fortnight before the full time. Three hours and a half after they commenced, and, ere the first stage of the labor was completed, I placed her under the influence of the chloroform, by moistening, with half a tea-spoonful of the liquid, a pocket handkerchief, rolled up into a funnel placed over her mouth and nostrils. In consequence of the evaporation of the fluid, it was once more renewed in about ten or twelve minutes. The child was expelled in about twenty-five minutes after the inhalation was begun. The mother subsequently remained longer soporose than commonly happen after ether. The squalling of the child did not,

* When used for surgical purposes, perhaps it would be found to be most easily given upon a handkerchief, gathered up into a cup-like form in the hand of the exhibitor, and with the open end of the cup placed over the nose and mouth of the patient. For the first inspiration or two, it should be held at the distance of half an inch or so from the face, and then more and more closely applied to it. To insure a rapid and perfect anæsthetic effect—more espially where the operation is to be severe—one or two teaspoonsful of the choloroform should be at once placed upon the hollow of the handkerchief, and immediately held to the face of the patient. Generally a snoring sleep supervenes: and when it does so, it is a perfect test of the superinduction of complete insensibility. But a patient may be quite anæsthetic without this system supervening.

†A young dentist who has himself had two teeth extracted lately—one under the influence of ether, and the other under the influence of chloroform—writes me the following ststement of the results: "About six months ago I had an upper molar tooth extracted whilst under the influence of ether, by Mr. Imlach. The inhalation was continued for several minutes before I presented the usual appearance of complete etherization; the tooth was then extracted; and, although I did not feel the least pain, yet I was conscious of the operation being performed, and was quite aware when the crash took place. Some days ago I required another molar extracted on account of tooth;ache, and this operation was again performed by the same gentleman. I inhaled the vapor of chloroform, half a drachm being poured upon a hankerchief for that purpose, and held to my nose and mouth. Insensibility took place in a few seconds; but I was so completely *dead* this time, that I was not in the very slightest degree aware of any thing that took place. The subsequent stupifying effects of the chloroform went off more rapidly than those of the ether; and I was perfectly well and able again for my work in a few minutes."

as usual, rouse her; and some minutes elapsed after the placenta was expelled, and after the child was removed by the nurse into another room, before the patient awoke. She then turned around and observed to me that she had "enjoyed a very comfortable sleep, and indeed required it, as she was so tired,* but would now be more able for the work before her." I evading entering into conversation with her, believing, as I have already stated, that the most complete possible quietude forms one of the principal secrets for the successful employment of either ether or chloroform. In a little time she again remarked that she was afraid her "sleep had stopped the pains." Shortly afterwards, her infant was brought in by the nurse from the adjoining room, and it was a matter of no small difficulty to convince the astonished mother that the labor was entirely over, and that the child presented to her was really her "own living baby."

Perhaps I may be excused from adding, that since publishing on the subject of ether inhalation in midwifery, seven or eight months ago,† and then for the first time directing the attention of the medical protession to its great use and importance in natural and morbid parturition, I have employed it, with few and rare exceptions, in every case of labor that I have attended; and with the most delightful results. And I have no doubt whatever, that some years hence the practice will be general. Obstetricians may oppose it, but I believe our patients themselves will force the use of it upon the profession.‡ I have never had the pleasure of watching over a series of better and more rapid recoveries; nor once witnessed any disagreeable result follow to either mother or child; whilst I have now seen an immense amount of maternal pain and agony saved by its employment. And I most conscientiously believe that the proud mission of the physician is distinctly twofold—namely, to alleviate human suffering, as well as preserve human life.

*Chemical Constitution of Chloroform.*—Formyle is the hypothetical radical of formic acid. In the red ant (*Formica rufa*) formic acid was first discovered, and hence its name. Gehlen pointed it out as a peculiar acid; and it was afterwards first artificially prepared by Doebereiner. Chemists have now devised a variety of processes, by which formic acid may be obtained from starch, sugar, and, indeed, most other vegetable substances.

A series chlorides of formyle are produced when chlorine and the hypochlorites are bronght to act on the chloride, oxide, and

---

*In consequence of extreme anxiety at the unfortunate result of her previous confinement, she had slept little or none for one or two nights, preceding the commencement of her present accouchement.

†See *Monthly Journal of Medical Science* for February, p. 539; for March, p. 718 and 721, and April, p. 794, &c.

‡I am told that the London physicians, with two or three exceptions only, have never yet employed ether inhalation in their midwifery practice. Three weeks ago, I was informed in a letter from Professor Montgomery of Dublin, that he believed that in that city, up to that date, it had not been used in a single case of labor.

hydrated oxide of methyle, (pyroxylic or wood spirit.) In the same way as formic acid may be artificially procured from substances which do not contain formyle ready formed, so also are the chlorides of this radical capable of being procured from substances which do not originally contain it.

Chloroform, chloroformyle, or the perchloride of formyle, may be made and obtained artificially by various processes—as by making milk of lime, or an aqueous solution of caustic alkali act upon chloral—by distilling alcohol,pyroxylic spirit, or acetone, with chloride of lime—by leading a stream of clorine gas into a solution of caustic potass in spirit of wine, &c. The preparation which I have employed, was made according to the formula of Dumas :

| | | |
|---|---|---|
| ℞ Chloride of lime in powder, | - | lb iv. |
| Water, | - - - - - | lb xii. |
| Rectified spirit, | - - - | f ℥ xii. |

"Mix in a capacious retort or still, and distil as long as a dense liquid, which sinks in the water with which it comes over, is produced."—(Gray's *Suppliment to the Pharmacopœia*, 1846, p. 633.)

The resulting perchloride of formyle consists of two atoms of carbon, one of hydrogen, and three of chlorine. Its specific gravity is much greater than the water, being as 1.480. It boils at 141°. The density of its vapor is 4.2. It is not inflammable ; nor changed by distillation with potassium, potash, sulphuric, or other acids. (See Turner's *Elements of Chemistry*, 8th edition, p. 1009 ; Gregory's *Outlines of Chemistry*, part ii. p. 401 ; Fownes' *Manual of Elementary Chemistry*, p. 419 ; Thomson's *Chemistry of Organic Bodies*, p. 312 ; Loewig's *Organische Chemie*, vol. i. p. 498.)

It is now well ascertained that three compound chemical bodies possess, when inhaled into the lungs, the power of superinducing a state of anæsthesia, or insensibility to pain in surgical operations, &c., namely, nitrous oxide, sulphuric ether, and perchloride of formyle. The following tabular view shows that these agents are entirely different from each other in their chemical constitution, and hence that their elementary composition affords no apparent clue to the explanation of their anæstheties :

| | Proportion of Nitrogen. | Of Oxygen. | Of Carbon. | Of Hydrogen. | Of Chlorine. |
|---|---|---|---|---|---|
| Nitrous Oxide, | 1 Atom. | 1 Atom. | | | |
| Sulphuric Ether, | | 1 Atom. | 4 Atoms. | 5 Atoms. | |
| Chloroform, | | | 2 Atoms. | 1 Atom. | 3 Atoms. |

It is perhaps not unworthy of remark, that when Soubeiran, Liebig, and Dumas engaged, a few years back, in those inquiries and experiments by which the formation and composition of chloroform was first discovered, their sole and only object was the investigation of a point in philosophical chemistry. They labored for the pure love and extension of knowledge. They had no idea that the substance to which they called the attention of their chemical brethren could or would be turned to any *practical* purpose, or that it possessed any physiological or therapeutic effects upon the animal economy. I mention this to show, that the *cui bona* argument against philosophical investigations on the ground that there may be at first no apparent practical benefit to be derived from them, has been amply refuted in this, as it had been in many other instances. For I feel assured, that the use of chloroform will soon entirely supersede the use of ether; and, from the facility and rapidity of its exhibition, it will be employed as an anæsthetic agent in many cases, and under many circumstances, in which ether would never have had recourse to. Here then we have a substance which, in the first instance, was merely interesting as a matter of scientific curiosity and research, becoming rapidly an object of intense importance, as an agent by which human suffering and agony may be annulled and abolished, under some of the most trying circumstances in which human nature is ever placed.

I have, up to this date, exhibited the chloroform to about fifty individuals. In not a single instance has the slightest bad result of any kind whatever occurred from its employment.

EDINBURGH, November 15, 1847.

